# Design of Financial Incentive Programs for Smoking Cessation: A Discrete Choice Experiment

**DOI:** 10.1093/ntr/ntac042

**Published:** 2022-02-15

**Authors:** Rachel J Breen, Matthew A Palmer, Mai Frandsen, Stuart G Ferguson

**Affiliations:** College of Health & Medicine, University of Tasmania, Tasmania, Australia; College of Health & Medicine, University of Tasmania, Tasmania, Australia; College of Health & Medicine, University of Tasmania, Tasmania, Australia; College of Health & Medicine, University of Tasmania, Tasmania, Australia

## Abstract

**Introduction:**

Financial incentive programs promote smoking cessation. However, the incentive amount which should be provided—and how this may interact with other program characteristics—is unknown. The objective of this study was to evaluate the influence of the design of incentive programs for smoking cessation on current smokers’ perceptions of programs and willingness to enroll.

**Method:**

An online discrete choice experiment was conducted amongst adult current smokers residing in the United Kingdom (*N* = 430). Hypothetical incentive programs were described using five attributes (incentive amount, incentive type, frequency of sessions, reward schedules, program location). Participants responded to a series of choice sets comprised of two hypothetical programs. For each set, participants selected their preferred program. They then specified whether they would enroll in their preferred program if it were available. Analyses also considered the effect of participant income on preferences.

**Results:**

Overall, participants preferred higher amounts over lower amounts, cash over vouchers, healthcare settings over workplaces, and consistent amounts over an escalating schedule. One session per week was the most preferred session frequency. Willingness to enroll increased quadratically with the incentive amount, although this increase slowed for higher amounts. Although middle- and high-income smokers preferred slightly higher amounts (cf. low-income participants), enrollment choices did not differ by income.

**Conclusion:**

The characteristics of incentive programs influence smokers’ perceptions of programs and willingness to enroll. Higher amounts may encourage greater enrollment rates, but there will likely be a ceiling point beyond which increasing the incentive amount does not meaningfully increase enrollments.

**Implications:**

There is increasing evidence incentive programs aid smoking cessation. Yet, the variety in previous program designs means how to best structure programs, including optimal incentive amount and the impact of the design on potential enrollment rates, remains unclear. This study suggests enrollments may be highest when incentive amounts are higher, rewards of a consistent amount in cash are provided, and sessions occur once per week in a healthcare setting. Although higher-income participants may desire higher incentive amounts compared to lower-income participants, this may not translate into differences in willingness to enroll.

## Introduction

The negative health consequences of smoking—including increased risk of cardiovascular disease, numerous cancers, and shorter life expectancy—have been extensively reported.^1,2^ Importantly for population health, quitting smoking reduces the risk of smoking-related morbidity and mortality.^1^ Despite the known benefits of quitting, only 40%–50% of smokers make a quit attempt each year.^3,4^ Of those, less than 10% will successfully quit.^4,5^ Additional methods are needed to encourage smokers to make quit attempts and sustain abstinence.

Financial incentive (FI) programs have been suggested as a method of addressing these goals. Within these programs, smoking status is monitored over a defined period, with individuals who demonstrate abstinence at various time points provided monetary rewards. Unlike more traditional smoking cessation programs, FI programs provide a salient and immediate reward for abstinence which can increase motivation to initiate quit attempts and, by rewarding behavior, encourage individuals to maintain abstinence. A recent Cochrane review^6^ suggests incentives are one of the most beneficial behavioral interventions for promoting smoking cessation. Such work^6,7^ supporting the efficaciousness of FI programs has led to an interest in these programs among policymakers and health service providers. However, the designs of previous programs have varied greatly.^8^ Variations include the FI amount provided, the type of FI (e.g., cash or vouchers), frequency of rewards, and location of implementation (e.g., pharmacies, workplaces, or antenatal clinics). This wide variety in previous designs means how to best structure programs remains unclear. This includes which program design(s) will be most desirable to smokers or result in the highest uptake. Furthermore, despite the evidence for the efficacy of FIs,^7^ some policymakers and the public remain skeptical about these programs. This Skepticism seems in part due to concerns about the seemingly high costs of FIs.^9^ Evidence for cost-efficient FIs (without sacrificing efficacy) is thus necessary to both ensure FI programs are well designed, and to address the concerns of potential implementers or users of these programs.

It has been suggested larger FI amounts will be more effective, based on the idea that FIs operate by increasing the benefits and value of smoking cessation.^10,11^ This notion is consistent with economic theory suggesting individuals will alter their behavior if the benefits outweigh the costs of change.^10^ Work in this area includes trials comparing relatively lower to higher FI amounts, typically finding greater abstinence rates for higher FI magnitudes.^12,13^ This provides foundational evidence that FI amounts differentially affect behavior. Yet values are commonly selected based on previously used schedules and budget/funding capacity. Hence, there is little evidence to suggest which amounts might most optimally balance effectiveness and value for money (funding allocation per enrollling participant), and a paucity of information to address public concerns about the justification for FI amounts. Although reviews—wherein a wider range of values can be considered—could be used to identify optimal amounts, such work only weakly supports an association between higher amounts and greater quit rates; a finding potentially due to the numerous variations in the design and target populations of previous studies.^8,14,15^ Our group has recently explored questions on FI amounts using experimental methods, wherein we observed an increasing quadratic trend in willingness to enroll in hypothetical programs as the FI amount increased.^16^ This work indicates there may be a ceiling point beyond which further increasing the FI amount fails to result in increased motivation to enroll. Further research is needed to explore the influence of reward amount, including how willingness to enroll may vary by the magnitude of the FI being offered.

In addition to FI amount, several other program characteristics may impact willingness to enroll. For example, policymakers and healthcare providers have theorized that the type of FI will be important, with smokers potentially preferring cash rewards compared to more inflexible rewards like vouchers or specific items.^17^ This idea is supported by research on FI programs for weight loss and exercise, which suggest more flexible FI types are associated with increased predicted enrollment in programs due to greater fungibility of the payment.^18,19^ The greater the perceived relevance or personal value of an incentive, the more appealing it will be to that individual. Hence individuals may value vouchers at less than their equivalent monetary worth, meaning voucher-based FIs may need to be of a greater value than cash-based FIs to ensure the same utility is afforded.^19^ In support of these theories, some research^20^ indicates vouchers are valued at only 80%–90% of their monetary amount.

Enrolment rates may similarly be impacted by where a program is located. The settings of previous FI work have varied widely, having included embedding programs into workplaces and a variety of healthcare centers.^7,8^ When considering the use of FIs in real-world settings, workplaces may be important implementation locations as employers could finance the incentives themselves, thereby reducing reliance on outside organizations and/or the healthcare system. Plausibly, smokers may prefer workplace programs due to the convenience. Alternatively, smokers may prefer healthcare settings like pharmacies or antenatal clinics as they associate these locations with greater quality or quantities of support. Yet the location preferred by most smokers is unclear.

Recipient characteristics like income level may also influence preferences for program design and willingness to enroll. This is consistent with economic theories including the law of diminishing marginal utility, which suggests the utility of an additional unit of money decreases as the individual’s current wealth increases.^21^ Lower-income smokers may therefore be more likely to enroll for smaller FIs than higher-income smokers, as the amount provides comparatively greater utility.^10^ However, research supporting this idea is mixed, with some studies^16,22^ suggesting no effect of income, while others^15,23^ indicate an effect at some timepoints or under some program designs.

Comparing multiple design aspects of FI programs through methods like randomized controlled trials would be time-consuming and costly. Instead, stated preference methods may be used to compare across multiple design characteristics more efficiently, to narrow the range of design options worth further investigation, and to guide future trials.^24^ Discrete choice experiments (DCEs) are a stated preference method that allows consideration of the perceived importance of multiple program attributes (characteristics of a program, e.g., FI type), and preferences for the levels of these attributes (the values the attribute may take, e.g., the levels of FI type could be cash or vouchers). Participants make a series of choices between specified options (e.g., select their preferred cessation program from two options with different designs) and by analyzing the pattern of responses across the series of choices, a good indication of what parameters shape individual’s preferences can be determined. This allows the comparison of potential programs in terms of a range of characteristics, including FI amounts, frequency of sessions, and rewards schedules. The use of DCEs has become increasingly common in health economics, with findings used to address policy questions and guide research that could seek to quantify results using revealed preference methods.^24^ The aim of this study was therefore to use a DCE to explore current smokers’ preferences for common FI program designs, and consider whether preferences varied between income groups.

## Method

### Overview

**Figure 1.**
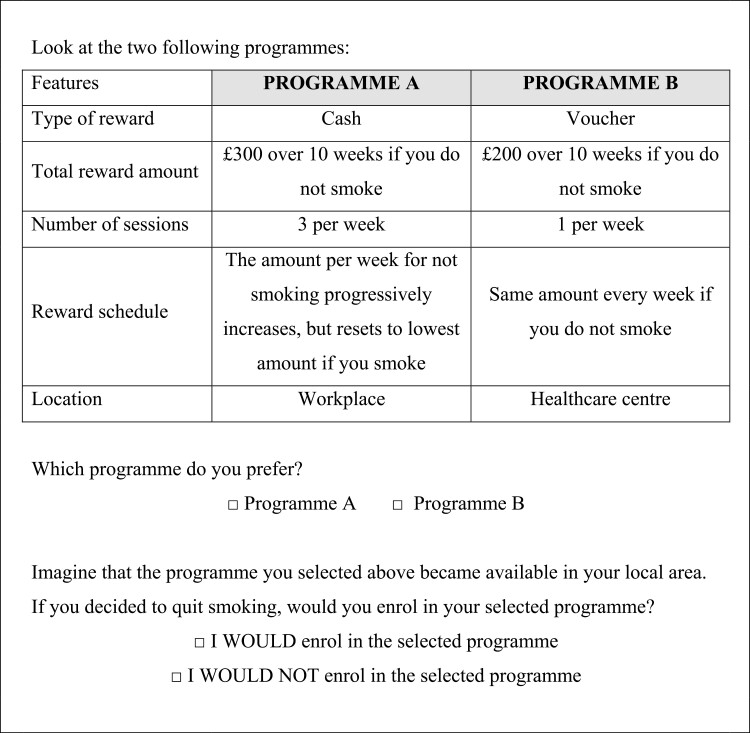
Example choice set.

The hypothetical programs within our DCE were described in terms of their attributes (e.g., FI type) and the levels of the attributes (the levels of FI type, e.g., cash or vouchers). Programs that differ in the levels of these attributes were paired together to form choice sets, and participants were asked to select which program from each set they most prefer. An example choice set is provided in Figure 1. We used a random utility model, wherein it was assumed the utility afforded by each program was dependent on the program design.^25,26^ Participants were therefore expected to select the program which provided the greatest expected utility from each choice set. By presenting participants with a series of choices wherein the characteristics of programs varied, the trade-offs between attributes could be assessed and their influence on preferences and potential uptake estimated.^25^

Participants completed the survey online. Ten choice sets each containing two, 10-week program options were presented one at a time. For each set, participants first selected the program they most preferred. They then indicated whether or not they would enroll in their preferred program if it were available. The participant then moved to the next choice set.

### Participants

Participants were UK residents aged 18 years or older who currently smoked. Current smoking was defined as regular smoking of tobacco products (e.g., cigarettes, cigars). Education was categorized as low (primary or secondary school, vocational training, trade apprenticeship), moderate (further education/training, college below degree level, some university), or high (completion of a university degree, post-graduate study).^27^ To test for differences in the perceptions of FI programs between income groups, recruitment was stratified by income. Based on OECD guidelines,^28^ participants were categorized as low- income (households earning < 75% of the median national average, or < £20,000), middle- income (75%–200%, or £20,000–£59,999), or high-income (>200% or > £60,000). It was intended to recruit 150 individuals per income group. The sample was not intended to be representative of the broader smoking population. The intended sample size (*N* = 450) was selected to exceed the minimum sample size recommended by commonly used rule-of-thumb estimates (e.g., 10 times the number of parameters)^29^ and was consistent with previous health-related DCEs.^30^

All participants were recruited from Prolific.co^31^, and completed the study online. Prolific.co is a research dedicated online platform used^32^ to recruit participants for predominantly psychological, social, and economic studies; see Palan and Schitter^33^ for an overview. All participants received financial compensation for their time, consistent with Prolific.co’s recommended minimum of £5 per hour. Recruitment and data collection were completed within August 2020. Ethics approval was obtained from the Tasmanian Social Science Human Research and Ethics Committee (H0017964).

### Attributes and Levels

Initial attributes and levels were drawn from a review of literature on previous FI programs^7,8^ and refined through team meetings. Five attributes were included in the final survey: FI amount, FI type, reward schedule, frequency of sessions, and program location (see Table 1). Seven FI amounts were presented. Each amount represented the maximum amount available for verified abstinence throughout the entire program. Upper and lower amounts were drawn from previous FI trials.^7^ Incentive type was either *cash* or *vouchers*. Although previous programs have also used lottery or deposit-based FIs, these forms of FIs vary on more than one dimension (e.g., lotteries differ from cash in both risk and fungibility); they were therefore excluded here to reduce the complexity of interpretation. To capture some of the variability in incentive schedules observed within previous trials, the reward schedule was described as either a *fixed schedule* (the same FI amount would be provided every week the individual did not smoke) or an *escalating schedule with reset contingency* (the amount per week for abstinence progressively increases for consecutive weeks of abstinence, but resets to lowest amount if the individual smokes). The sessions attribute denoted the frequency of sessions: *one/fortnight, one/week, two/week, or three/week*. All programs lasted a total of 10 weeks. Finally, there were two levels of location (a generic *Healthcare center* or the individual’s *workplace*), reflecting real-world settings wherein FI programs have previously been implemented.

**Table 1.** Discrete Choice Experiment Attributes and Levels

Attribute	Levels
Incentive amount	£50; £100; £200; £350; £500; £750; £1,000
Incentive type	Voucher; Cash
Reward schedule	Escalating with reset contingency (the amount per week progressively increases, but resets to first amount if you smoke);
	Fixed (same amount every week that you do not smoke)
Frequency of sessions	One/fortnight; One/week; Two/week; Three/week
Program location	Healthcare center; Workplace

### Survey Design

Given the number of attributes and levels included, a full factorial design was deemed impractical. A fractional factorial design with zero priors (utility neutral design)^34^ was therefore constructed using JMP (version 14)^35^ and checked for level balance, minimal overlap, and orthogonality. This produced 30 choice sets, which were divided into three blocks comprising of 10 sets per block (see Supplementary Material for choice sets). Participants completed one of the three blocks to ensure cognitive burden was minimized.^36^ Assignment to blocks was stratified to ensure approximately equal numbers of participants from each income group were assigned to each block (150 participants total intended per block).

### Procedure

Within the online survey, participants were first asked sociodemographic and smoking behavior questions. Instructions on how to complete the discrete choice task, a detailed description of all attributes and levels, and an example program were then presented. All participant then completed a dominance check to ensure they had understood the choice task, were paying attention, and as an indication of rational choice behavior.^37^ This consisted of one additional choice set wherein one program (the dominant option) was more appealing than the other in all ways. Participants were expected to choose the dominant option. Participants then completed the 10 choices sets from their assigned block. Each choice set was viewed one at a time. For each set, participants first selected their preferred program. They then indicated whether or not they would enroll in their preferred program if it were available to them in their local area (binary response; see example choice set in Figure 1). Progression through the choice sets was self-paced; participants clicked next to move to the next choice set. They were unable to return to previous choice sets.

### Data Reduction and Analyses

Eight respondents completed only the consent procedures, and two additional respondents started but did not complete the survey. There is no information available on why this occurred (e.g., technical issues, no longer interested). These participants were removed from the sample and additional recruitment was undertaken to obtain the prespecified sample size.

Once data collection was complete, three participants were identified as nontraders (always selected Program A over Program B), suggesting they had failed to attend to the attributes; these participants were removed from the sample. Additionally, 15 individuals who failed the dominance check (i.e., did not select the dominant option) were identified and removed from analyses. We were most interested in how to optimize program designs and willingness to enroll among individuals who would consider using these programs, and reasoned that participants who rejected even very lucrative amounts (£1000) were unlikely to entertain enrolling in real FI programs. Hence, participants who would not enroll in any program (*n* = 12) were excluded. Analyses confirmed removing these individuals did not alter the pattern of effects. The final evaluable sample was *N* = 430.

Data were analyzed using mixed logit models, which were constructed in R using the “mlogit” package. Mixed logits were selected over other models (e.g., multinomial logit) to account for preference heterogeneity among participants. Furthermore, as opposed to standard logistic models, this approach allows consideration of the fact participants respond to multiple independent choice sets. Our study design effectively allowed participants to rank the different hypothetical programs in order of preference. This design and statistical approach allow us to assess the effect of any given program characteristic independent of the other characteristics. For detailed information on these models see Croissant^38^ and Train^39^.

The FI amount was a continuous variable (rescaled to assist with interpretation, but this has no effect on the magnitude of the relationships). Other attributes were effects coded and included as random parameters with a normal distribution. With effects coding, the mean effect of all levels of an attribute is zero, and *p* values indicate the significance of the difference between the estimated value and the mean effect of the attribute. The estimate for the reference (omitted) level is the negative sum of the estimates for the other levels of that attribute.^25^ An alternative-specific constant for the would not enroll option was included to adjust the attribute coefficients to account for people opting out.^40^ The final model was used to consider participants’ preferences for program characteristics, and differences between income groups. Significant standard deviations indicate preference heterogeneity. A simpler model without interactions (participant income) was also constructed; this produced very similar results (see Supplementary Material).

Marginal willingness to accept indicates the total monetary amount offered throughout the program associated with changes in attribute levels.^41^ This was calculated using estimates from the final model by dividing the estimated coefficients of the nonmonetary attributes by the negative of the FI amount coefficient. The Krinsky–Robb method was used to calculate confidence intervals.^41^

Willingness to enroll was first discussed regarding the frequency at which participants would hypothetically enroll in their preferred program from each choice set. Changes in estimated program uptake across amounts, and secondly across other attributes, were then determined by calculating the choice probabilities using estimates from the model without interactions (see Supplementary Material).^25^ Raw data and r code are available from the University of Tasmania’s data portal (https://dx.doi.org/10.25959/ccv4-4q20).

## Results

### Participants

On average, participants were 37.47 years old (SD = 12.27) and approximately 56.51% were female. More than half of participants had made a quit attempt in the past year (62.09%) and intended to quit smoking in the next 6 months (57.67%). The sample was comparable to the broader population of UK smokers regarding age and gender distributions.^42^ However, there were some education level differences, with a higher proportion of our sample having completed a university degree (compared to the broader smoking population). The demographic and smoking characteristics of participants by income group are displayed in the Supplementary Material.

### Preferences for Attributes

Model results are summarized in Table 2. Higher coefficients indicate a greater preference for an attribute level. Negative coefficients indicate disutility. All attributes were important to preferences. Across participants, people preferred higher amounts over lower amounts, cash over vouchers, healthcare settings over workplaces, and consistent amounts over an escalating schedule with a reset contingency. One session per week was the most preferred frequency, followed by one session per fortnight, and then two per week, although the differences in preferences for these frequencies were minimal. Three sessions per week were the least preferred. As observed from the interaction terms, compared to low-income participants, middle- and high-income participants preferred slightly higher FI amounts (*p* = .021 and *p* = .038, respectively).

**Table 2.** Mixed Logit Estimation Results

Predictors	Estimate (SE)	SD (SE)	WTA (95% CI)
Amount	0.046 (0.002)***	—	—
Cash	0.331 (0.031)***	0.416 (0.052)***	−74.96 (−60.83, −90.47)
Vouchers	−0.331		
Healthcare center	0.124 (0.031)***	0.549 (0.047)***	−28.16 (−42.61, −14.64)
Workplace	−0.124		
Consistent	0.067 (0.031)*	0.120 (0.113)	−15.14 (−1.51, −29.12)
Escalating	−0.067		
1 session per fortnight	0.221 (0.056)***	0.393 (0.100)***	−49.94 (−25.46, −76.25)
1 session per week	0.273(0.059)***	0.290 (0.153)*	−61.70 (−35.52, −89.09)
2 sessions per week	0.114 (0.063)	0.022 (0.381)	−25.79(1.90, −53.30)
3 sessions per week	-0.608		
No program^a^	1.102 (0.137)***		−249.48 (−203.49, −290.22)
Amount*middle income	0.065 (0.003)*		
Amount*high income	0.007 (0.003)*		
No program*middle income	0.067 (0.162)		
No program*high income	0.363 (0.187)		

*Note:* Observations (N*tasks) = 4,300. Log-likelihood = −3,132.4.

SE = standard error; SD = standard deviation; CI = confidence intervals; WTA = willingness to accept.

^a^a constant = 1 if “would not enrol in a program” was selected or = 0 if the participant would enroll in their preferred program.

*p < .05; **p < .01; ***p < .001.

The average FI amount associated with changing attribute levels from the reference level is displayed in Table 2. When holding the program design constant, the amount necessary for participants to be indifferent between enrolling in a program and not enrolling was approximately £248 (95%CI = £202.80–£290.78) across the program.

### Willingness to Enrol

Responses to the willingness to enroll questions were first used to provide insight into the general willingness to enroll in FI programs. Overall, 45.02% (*n =* 199) of participants were willing to enroll in all programs. Conversely, 2.71% of participants (*n =* 12) would not enroll in any of the presented programs (these participants were not included in other analyses). On average, the proportion of “would enroll” responses across all participants and choice sets was 81.22%, reflecting a very high potential enrollment rate. Based on the model (see Table 2), differences in hypothetical enrolment between low- and middle-income groups were nonsignificant (*p* = .680), indicating both groups would be similarly willing to enroll in a program with a specific design. Although there was a trend toward high-income participants being more likely to opt-out than low-income participants, this did not reach significance (*p* = .052). Low-income participants opted out in 17.04% of choice sets, while high-income participants opted out in 18.28% of choices.

**Figure 2.**
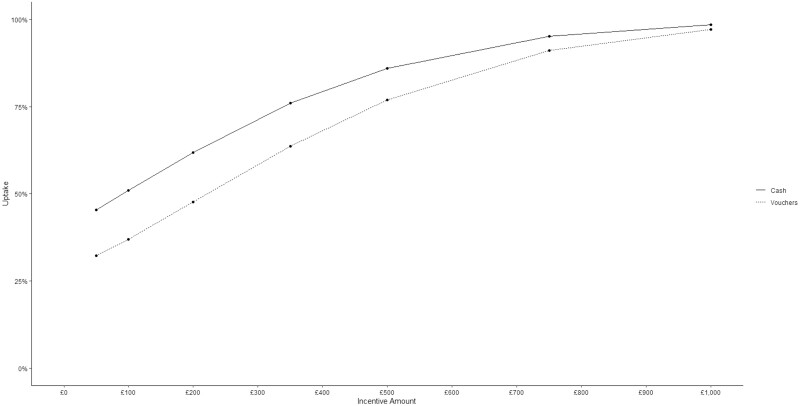
Predicted program uptake across incentive amounts.

Differences in the estimated uptake for the most preferred program (cash rewards, consistent amounts, one session per week, and within a healthcare center) across FI amounts are summarized in Figure 2. As differences in enrolment by income group were nonsignificant, uptake estimates were derived from the simpler model (see Supplementary Material). As expected, the estimated uptake increased across amounts, with 45.36% predicted to enroll for a total of £50 across the program, increasing up to 98.86% for £1,000. Interestingly, predicted uptake followed a concave quadratic trend. Doubling the FI amount from £500 to £1,000 increased predicted uptake by only 10.62%.

Changing from cash to voucher rewards while retaining other attribute levels lowered the predicted uptake, although the size of this difference decreased as the FI amount increased (see Figure 2). Please see https://tinyurl.com/dce-financial-incentives for an interactive figure (shiny app)^43^ displaying variations in estimated uptake for other combinations of program characteristics.

## Discussion

We here considered how current smokers’ preferences for FI-based smoking cessation programs vary depending on the FI amount and program design. Results suggest that to maximize smokers liking of FI programs and potential enrolment rates, programs could use higher FI amounts, provide rewards of a consistent amount per week in cash, offer sessions once per week, and provide programs in healthcare settings. The observed preferences for the levels of attributes were largely consistent with previous research; preferences for higher FI amounts^19,40^ and for more flexible payments like cash (cf. vouchers)^17,19^ have been observed when considering FI programs for behaviors including drug use and exercise. Although workplaces could prove an important implementation location because employers could finance incentives themselves rather than relying on the healthcare system, participants in our study preferred healthcare settings. A slight preference for one session per week over one session per fortnight may be explained by some participants perceiving they would not receive sufficient support if sessions were fortnightly.

The FI amount required has been suggested to differ by recipient income, such that lower-income smokers may be motivated by one FI amount, but the amount may need to increase comparatively to income to ensure the same viability.^10^ Consistent with this idea, results here suggested middle- and high-income participants preferred slightly higher amounts than low-income smokers. However, differing preferences for FI amounts did not translate into significant differences in predicted enrolment. This is comparable to previous work by our group,^16^ wherein high-income (cf. low-income) current smokers viewed FI programs as less appealing, but remained equally willing to enroll in programs. These findings add support for the use of FI programs in practice, as it suggests amounts might produce similar enrolment rates across income groups.

Across choice sets, participants would enroll in their preferred program 81.22% of the time, suggesting a high level of interest and potential willingness to enroll in FI programs for smoking cessation. As was expected based on previous research,^13,16^ the estimated enrolment rates increased across FI amounts. This increase largely followed a concave quadratic trend. While not investigated here, such a quadratic trend will likely contain a peak or ceiling point beyond which the increasing trend levels off. This point may indicate an incentive value beyond which increasing the amount will not meaningfully benefit enrolment rates. This finding is consistent with some previous work^40^ investigating incentivizing health behaviors other than smoking, and with experimental work by our group wherein current smokers’ perceptions of FI programs increased quadratically with the FI amount, up to a ceiling point.^16^ Varying other elements of the program design also influenced estimated uptake. However, the magnitude of the differences in estimated uptake produced by varying other design characteristics decreased as the FI amount increased, reaffirming the importance of the amount, and suggesting higher amounts may compensate for less desirable designs.

The method used within this study was a low-cost way of indicating what people may value in FI-based smoking cessation programs. It provides the groundwork for future studies which further investigate the optimal design of programs. Although the choices made within hypothetical scenarios may differ from those made in real settings, some research^44^ does suggest DCEs on some health programs can reflect real-world decisions reasonably well. Relatedly, we here measured interest in enrolment as opposed to the effectiveness of amount at achieving outcomes once individuals enroll. This may mean that, for example, while participants here preferred consistent rewards, variable reinforcement and escalating schedules could better promote cessation outcomes.^45^ Rates of enrolment may similarly differ from rates of engagement with program content, as observed in some^46^ previous real-world programs. This was not investigable within this study. Hence, although results presented here should still be interpreted with caution, they may be indicative of what smokers would value regarding program design when considering whether to enroll.

There are some limitations of the work. The included sample was not intended to be representative of the broader smoking population, and indeed had a higher level of educational attainment. However, previous work has shown no clear evidence for the effect of education. For example, while some^47^ have found lower support for FI use among individuals with lower levels of education, several others^48,49^ show no effect of education. Furthermore, reviews^23^ suggest no differential effects of FIs on cessation outcomes by education level. Relatedly, it is possible demographic characteristics other than income might moderate an individual’s preferences; something future studies may wish to explore. Furthermore, the additional supports used in some previous FI trials (e.g., counseling or pharmacotherapy) were not considered. The program length was also consistent across all choice options, as varying the length would have increased task complexity. These factors will probably also affect program appraisals and therefore enrolments. Testing in alternative populations and other locations is also necessary, as factors including purchasing power may differ. Using the evidence base provided here, future investigations into the design of programs could consider these additional points, and subsequently test FI levels in real-world conditions.

## Conclusion

Additional methods are needed to encourage smokers to make quit attempts and sustain abstinence. While FI programs can help achieve these goals, clarity around the best program design is necessary to ensure smokers find programs desirable and will enroll, and to facilitate translatability into real-world settings. Our results suggest enrolment rates may be highest when programs use higher FI amounts, provide rewards of a consistent amount in cash, offer sessions once per week, and provide programs in healthcare settings. Although higher-income participants desired slightly higher amounts than lower-income participants, differences in hypothetical willingness to enroll were not significant.

## Supplementary Material

A Contributorship Form detailing each author’s specific involvement with this content, as well as any supplementary data, are available online at https://academic.oup.com/ntr.

ntac042_suppl_Supplementary_Material

ntac042_suppl_Supplementary_Taxonomy_Form

## Data Availability

The data underlying this article are available in the University of Tasmania’s Research Data Portal at https://dx.doi.org/10.25959/ccv4-4q20.

## References

[1] Jha P , Ramasundarahettige C, Landsman V, et al 21st-century hazards of smoking and benefits of cessation in the United States. N Engl J Med. 2013;368(4):341–350. 23343063 10.1056/NEJMsa1211128

[2] Gakidou E , Afshin A, Abajobir AA, et al Global, regional, and national comparative risk assessment of 84 behavioural, environmental and occupational, and metabolic risks or clusters of risks, 1990–2016: a systematic analysis for the Global Burden of Disease Study 2016. Lancet. 2017;390(10100):1345–1422. 28919119 10.1016/S0140-6736(17)32366-8 PMC5614451

[3] Borland R , Partos TR, Yong H-H, Cummings KM, Hyland A. How much unsuccessful quitting activity is going on among adult smokers? Data from the International Tobacco Control Four Country cohort survey. Addiction. 2012;107(3):673–682. 21992709 10.1111/j.1360-0443.2011.03685.x PMC3909986

[4] Babb S , Malarcher A, Schauer G, Asman K, Jamal A. Quitting smoking among adults – United States, 2000–2015. CDC- Morbilty Mortal Wkly Rep. 2017;65(52):1457–1464. 10.15585/mmwr.mm6552a1 28056007

[5] Australian Institute of Health and Welfare. The National Drug Strategy Household Survey 2016: Detailed Findings. Australian Institute of Health and Welfare; 2017. https://www.aihw.gov.au/getmedia/15db8c15-7062-4cde-bfa4-3c2079f30af3/21028a.pdf.aspx?inline=true

[6] Hartmann-Boyce J , Livingstone-Banks J, Ordóñez-Mena JM, et al. Behavioural interventions for smoking cessation: an overview and network meta-analysis. Cochrane Database Syst Rev. 2021;CD013229. doi:10.1002/14651858.CD013229.pub2 PMC11354481 33411338

[7] Notley C , Gentry S, Livingstone-Banks J, et al Incentives for smoking cessation (Review). Cochrane Database Syst Rev. 2019;7:CD004307. doi:10.1002/14651858.CD004307.pub6 PMC6635501 31313293

[8] Breen RJ , Ferguson SG, Palmer MA. Higher incentive amounts do not appear to be associated with greater quit rates in financial incentive programmes for smoking cessation. Addict Behav. 2020;110:106513. doi:10.1016/j.addbeh.2020.106513 32590220

[9] Wen X , Higgins ST, Xie C, Epstein LH. Improving public acceptability of using financial incentives for smoking cessation during pregnancy: a randomized controlled experiment. Nicotine Tob Res. 2016;18(5):913–918. 26385928 10.1093/ntr/ntv204

[10] Scott A , Schurer S. Financial Incentives, Personal Responsibility and Prevention. Discussion paper commissioned by the National Health and Hospitals Reform Commission, Australian Government; 2008. https://www.researchgate.net/publication/228798450_Financial_incentives_personal_responsibility_and_prevention

[11] Sosa-Rubí SG , Galárraga O. Economic Incentives, Risk Behaviors, and HIV. In: Oxford Research Encyclopedia of Economics and Finance. Oxford University Press; 2019:1–38. doi:10.1093/acrefore/9780190625979.013.249

[12] Higgins ST , Heil SH, Dantona R, et al Effects of varying the monetary value of voucher-based incentives on abstinence achieved during and following treatment among cocaine-dependent outpatients. Addiction. 2007;102(2):271–281. 17222282 10.1111/j.1360-0443.2006.01664.x

[13] Packer RR , Howell DN, McPherson S, Roll JM. Investigating reinforcer magnitude and reinforcer delay: a contingency management analog study. Exp Clin Psychopharmacol. 2012;20(4):287–292. 22686494 10.1037/a0027802

[14] Giles EL , Robalino S, McColl E, Sniehotta FF, Adams J. The effectiveness of financial incentives for health behaviour change: systematic review and meta-analysis. PLoS One. 2014;9(3):e90347. 24618584 10.1371/journal.pone.0090347 PMC3949711

[15] Mantzari E , Vogt F, Shemilt I, et al Personal financial incentives for changing habitual health-related behaviors: a systematic review and meta-analysis. Prev Med (Baltim). 2015;75:75–85. 10.1016/j.ypmed.2015.03.001 PMC4728181 25843244

[16] Breen RJ , Ferguson SG, Palmer MA. Smokers’ perceptions of incentivised smoking cessation programmes: examining how payment thresholds change with income. Nicotine Tob Res. 2021;23(9):1–10. doi:10.1093/ntr/ntab031 33621322

[17] Giles EL , Sniehotta FF, McColl E, Adams J. Acceptability of financial incentives for health behaviour change to public health policymakers: a qualitative study. BMC Public Health. 2016;16(1):1–11. 27633661 10.1186/s12889-016-3646-0 PMC5025536

[18] Hashemi A , You W, Boyle KJ, et al Identifying financial incentive designs to enhance participation in weight loss programs. J Obes Weight Loss Ther. 2015;05(01):1–8.

[19] Farooqui MA , Tan YT, Bilger M, Finkelstein EA. Effects of financial incentives on motivating physical activity among older adults: results from a discrete choice experiment. BMC Public Health. 2014;14(1):1–9. doi:10.1186/1471-2458-14-141 PMC3933254 24512102

[20] Rosado J , Sigmon SC, Jones HE, Stitzer ML. Cash value of voucher reinforcers in pregnant drug-dependent women. Exp Clin Psychopharmacol. 2005;13(1):41–47. 15727502 10.1037/1064-1297.13.1.41

[21] Greene J , Baron J. Intuitions about declining marginal utility. J Behav Decis Mak. 2001;14(3):243–255.

[22] López-núñez C , Secades-villa R, Peña-suárez E, et al Income levels and response to contingency management for smoking cessation. Randomized Controlled Trial. 2017;52(7):6084. doi:10.1080/10826084.2016.1264973 28426355

[23] Haff N , Patel MS, Lim R, et al The role of behavioral economic incentive design and demographic characteristics in financial incentive-based approaches to changing health behaviors: a meta-analysis. Am J Heal Promot. 2015;29(5):314–323. 10.4278/ajhp.140714-LIT-333 25928816

[24] Louviere JJ , Lancsar E. Choice experiments in health: the good, the bad, the ugly and toward a brighter future. Heal Econ Policy Law. 2009;4(4):527–546. 10.1017/S1744133109990193 19715635

[25] Louviere JJ , Swait J, Hensher DA. Stated Choice Methods: Analysis and Applications. Cambridge: Cambridge University Press; 2000.

[26] Horowitz J , Keane M, Bolduc D, et al Advances in random utility models report of the workshop on advances in random utility models duke invitational symposium on choice modeling behavior. Mark Lett. 1994;5(4):311–322. http://mpra.ub.uni-muenchen.de/53026/.

[27] Hitchman SC , Brose LS, Brown J, Robson D, McNeill A. Associations between E-Cigarette type, frequency of use, and quitting smoking: findings from a longitudinal online panel survey in Great Britain. Nicotine Tob Res. 2015;17(10):1187–1194. 25896067 10.1093/ntr/ntv078 PMC4580313

[28] OECD. Under Pressure: The Squeezed Middle Class. Paris: OECD Publishing; 2019. doi:10.1787/689afed1-en

[29] Donnan JR , Johnston K, Chibrikov E, et al Capturing adult patient preferences toward benefits and risks of second-line antihyperglycemic medications used in type 2 diabetes: a discrete choice experiment. Can J Diabetes. 2020;44(1):6–13. 31311729 10.1016/j.jcjd.2019.04.014

[30] Soekhai V , de Bekker-Grob EW, Ellis AR, Vass CM. Discrete choice experiments in health economics: past, present and future. PharmacoEcon. 2019;37(2):201–226. 10.1007/s40273-018-0734-2 PMC6386055 30392040

[31] Prolific. First released 2014. Oxford, UK; 2019. https://app.prolific.co/

[32] Blackwell AKM , De-Loyde K, Brocklebank LA, et al Tobacco and electronic cigarette cues for smoking and vaping: an online experimental study. BMC Res Notes. 2020;13(1):1–6. 31941548 10.1186/s13104-020-4899-3 PMC6964108

[33] Palan S , Schitter C. Prolific.ac—a subject pool for online experiments. J Behav Exp Financ. 2018;17:22–27.

[34] Kessels R , Jones B, Goos P. An improved two-stage variance balance approach for constructing partial profile designs for discrete choice experiments. Appl Stoch Model Bus Ind. 2015;31(5):626–648.

[35] JMP®, Version 14. Cary, NC: SAS Institute Inc., 1989–2020.

[36] Bech M , Kjaer T, Lauridsen J. Does the number of choice sets matter? Results from a web survey applying a discrete choice experiment. Health Econ. 2011;20(3):273–286. 20143304 10.1002/hec.1587

[37] Tervonen T , Schmidt-Ott T, Marsh K, et al Assessing rationality in discrete choice experiments in health: an investigation into the use of dominance tests. Value Heal. 2018;21(10):1192–1197. 10.1016/j.jval.2018.04.1822 30314620

[38] Croissant Y . Estimation of random utility models in R: The mlogit Package. J Stat Softw 2020;95(11):1–41.

[39] Train K. Discrete Choice Methods with Simulation. New York: Cambridge University Press; 2003. doi:10.1017/cbo9780511805271.006

[40] Marti J , Bachhuber M, Feingold J, Meads D, Richards M, Hennessy S. Financial incentives to discontinue long- term benzodiazepine use: a discrete choice experiment investigating patient preferences and willingness to participate. BMJ Open. 2017;7(10):1–9. doi:10.1136/bmjopen-2017-016229 PMC5640034 28988167

[41] Hole AR . A comparison of approaches to estimating confidence intervals for willingness to pay measures. Health Econ. 2007;16(8):827–840. 17238222 10.1002/hec.1197

[42] Office for National Statistics. Adult Smoking Habits in the UK: 2019. ONS; 2019. https://www.ons.gov.uk/peoplepopulationandcommunity/healthandsocialcare/healthandlifeexpectancies/bulletins/adultsmokinghabitsingreatbritain/2019

[43] Chang W , Cheng J, Xie Y, McPherson J. Shiny: Web Application Framework for R. Version 1.6.0; 2021. https://cran.r-project.org/package=shiny

[44] de Bekker-Grob EW , Swait JD, Kassahun HT, et al Are healthcare choices predictable? The impact of discrete choice experiment designs and models. Value Heal. 2019;22(9):1050–1062. 10.1016/j.jval.2019.04.1924 31511182

[45] Sigmon SC , Patrick ME. The use of financial incentives in promoting smoking cessation. Prev Med (Baltim). 2012;55:S24–S32 10.1016/j.ypmed.2012.04.007 PMC3411852 22525802

[46] Halpern SD , Harhay MO, Saulsgiver K, Brophy C, Troxel AB, Volpp KG. A pragmatic trial of E-cigarettes, incentives, and drugs for smoking cessation. N Engl J Med. 2018;378(24):2302–2310. 29791259 10.1056/NEJMsa1715757

[47] Hoddinott P , Morgan H, MacLennan G, et al Public acceptability of financial incentives for smoking cessation in pregnancy and breast feeding: a survey of the British public. BMJ Open. 2014;4(7):e0055241–ee005524. 10.1136/bmjopen-2014-005524 PMC4120368 25037645

[48] Giles EL , Becker F, Ternent L, et al Acceptability of financial incentives for health behaviours: a discrete choice experiment. PLoS One. 2016;11(6):e01574031–e01574019. 10.1371/journal.pone.0157403 PMC4912063 27314953

[49] Bigsby E , Seitz HH, Halpern SD, Volpp KG, Cappella JN. Estimating acceptability of financial health incentives. Heal Educ Behav. 2017;44(4):513–518. 10.1177/1090198116664072 27535320

[50] Borland R , Yong HH, O’Connor RJ, Hyland A, Thompson ME. The reliability and predictive validity of the heaviness of smoking index and its two components: findings from the International Tobacco Control Four Country Study. Nicotine Tob Res. 2010;12(Suppl. 1):45–50. 10.1093/ntr/ntq038 PMC3307335 20889480

