# Supplementary material for: Design of Financial Incentive Programs for Smoking Cessation: A Discrete Choice Experiment
Source: Nicotine Tob Res. 2022 Feb 15;24(10):1661–8. doi: 10.1093/ntr/ntac042 (PMC9575978; doi:10.1093/ntr/ntac042)
Supplement: ntac042_suppl_Supplementary_Material [file ntac042_suppl_supplementary_material.docx]

**Supplementary Material**

**Table S1: Choice sets**

| **Block** | **Choices pair indicator** | **Type** | **Amount** | **Schedule** | **Session** | **Location** |
| --- | --- | --- | --- | --- | --- | --- |
| 1 | 1 | Cash | £750 | Consistent | 2 every week | Healthcare |
| 1 | 1 | Voucher | £200 | Escalate | 1 every 2 weeks | Workplace |
| 1 | 2 | Cash | £100 | Consistent | 1 every 2 weeks | Healthcare |
| 1 | 2 | Cash | £750 | Escalate | 1 every week | Workplace |
| 1 | 3 | Voucher | £200 | Consistent | 3 every week | Workplace |
| 1 | 3 | Cash | £350 | Escalate | 1 every week | Healthcare |
| 1 | 4 | Voucher | £750 | Escalate | 1 every 2 weeks | Healthcare |
| 1 | 4 | Cash | £750 | Consistent | 3 every week | Workplace |
| 1 | 5 | Cash | £200 | Consistent | 1 every week | Healthcare |
| 1 | 5 | Voucher | £1,000 | Consistent | 1 every 2 weeks | Workplace |
| 1 | 6 | Cash | £350 | Consistent | 2 every week | Workplace |
| 1 | 6 | Cash | £50 | Escalate | 1 every 2 weeks | Healthcare |
| 1 | 7 | Voucher | £750 | Consistent | 1 every week | Healthcare |
| 1 | 7 | Cash | £350 | Escalate | 3 every week | Workplace |
| 1 | 8 | Voucher | £100 | Consistent | 2 every week | Healthcare |
| 1 | 8 | Voucher | £1,000 | Escalate | 1 every 2 weeks | Workplace |
| 1 | 9 | Cash | £50 | Escalate | 1 every 2 weeks | Healthcare |
| 1 | 9 | Voucher | £350 | Consistent | 2 every week | Workplace |
| 1 | 10 | Cash | £1,000 | Escalate | 1 every week | Healthcare |
| 1 | 10 | Voucher | £1,000 | Consistent | 3 every week | Workplace |
| 2 | 1 | Cash | £50 | Escalate | 3 every week | Workplace |
| 2 | 1 | Voucher | £350 | Escalate | 1 every 2 weeks | Healthcare |
| 2 | 2 | Voucher | £200 | Escalate | 1 every week | Healthcare |
| 2 | 2 | Cash | £350 | Consistent | 1 every 2 weeks | Healthcare |
| 2 | 3 | Cash | £1,000 | Escalate | 2 every week | Healthcare |
| 2 | 3 | Voucher | £1,000 | Consistent | 1 every week | Workplace |
| 2 | 4 | Cash | £200 | Consistent | 2 every week | Healthcare |
| 2 | 4 | Voucher | £200 | Escalate | 1 every 2 weeks | Workplace |
| 2 | 5 | Voucher | £100 | Consistent | 1 every 2 weeks | Workplace |
| 2 | 5 | Cash | £350 | Consistent | 3 every week | Healthcare |
| 2 | 6 | Cash | £500 | Escalate | 2 every week | Workplace |
| 2 | 6 | Voucher | £200 | Consistent | 1 every 2 weeks | Healthcare |
| 2 | 7 | Voucher | £100 | Consistent | 1 every week | Healthcare |
| 2 | 7 | Cash | £750 | Escalate | 1 every 2 weeks | Healthcare |
| 2 | 8 | Voucher | £50 | Consistent | 3 every week | Workplace |
| 2 | 8 | Cash | £200 | Escalate | 3 every week | Healthcare |
| 2 | 9 | Voucher | £750 | Consistent | 1 every week | Healthcare |
| 2 | 9 | Cash | £500 | Escalate | 2 every week | Healthcare |
| 2 | 10 | Voucher | £200 | Consistent | 1 every week | Healthcare |
| 2 | 10 | Voucher | £750 | Escalate | 3 every week | Workplace |
| 3 | 1 | Cash | £100 | Escalate | 1 every 2 weeks | Workplace |
| 3 | 1 | Voucher | £100 | Consistent | 3 every week | Healthcare |
| 3 | 2 | Voucher | £50 | Escalate | 3 every week | Workplace |
| 3 | 2 | Cash | £750 | Consistent | 3 every week | Healthcare |
| 3 | 3 | Cash | £200 | Consistent | 1 every week | Workplace |
| 3 | 3 | Voucher | £350 | Escalate | 1 every week | Healthcare |
| 3 | 4 | Cash | £500 | Escalate | 3 every week | Workplace |
| 3 | 4 | Voucher | £1,000 | Escalate | 1 every 2 weeks | Healthcare |
| 3 | 5 | Voucher | £500 | Escalate | 1 every 2 weeks | Healthcare |
| 3 | 5 | Cash | £350 | Escalate | 2 every week | Workplace |
| 3 | 6 | Cash | £500 | Consistent | 2 every week | Workplace |
| 3 | 6 | Cash | £1,000 | Escalate | 3 every week | Healthcare |
| 3 | 7 | Cash | £200 | Consistent | 1 every week | Healthcare |
| 3 | 7 | Voucher | £500 | Consistent | 3 every week | Workplace |
| 3 | 8 | Cash | £1,000 | Escalate | 2 every week | Workplace |
| 3 | 8 | Voucher | £100 | Consistent | 2 every week | Healthcare |
| 3 | 9 | Cash | £500 | Escalate | 1 every 2 weeks | Workplace |
| 3 | 9 | Voucher | £100 | Consistent | 3 every week | Workplace |
| 3 | 10 | Cash | £50 | Consistent | 1 every week | Healthcare |
| 3 | 10 | Voucher | £50 | Escalate | 2 every week | Workplace |

| **Table S2:** Demographic characteristics and smoking history | | | | |
| --- | --- | --- | --- | --- |
|  | Total  sample  N = 430 | Low-  income  n = 115 | Middle-income  n = 204 | High-  income  n = 111 |
| Age | 37.47 (12.27) | 39.59 (14.16) | 37.01 (11.19) | 36.14 (10.94) |
| Gender (%) |  |  |  |  |
| Female | 56.51 | 68.70 | 54.41 | 47.75 |
| Education (%) |  |  |  |  |
| Low | 21.40 | 33.04 | 18.14 | 13.51 |
| Moderate | 38.84 | 41.74 | 43.14 | 27.93 |
| High | 39.76 | 25.22 | 37.75 | 58.56 |
| Years smoked | 17.91 (12.74) | 20.70 (14.72) | 17.96 (12.34) | 14.93 (10.76) |
| Intention to quit (%) |  |  |  |  |
| Within next 6 months | 57.67 | 47.83 | 61.76 | 60.36 |
| Within next 12 months/never | 42.33 | 52.17 | 38.24 | 39.64 |
| Quit attempts in past year (%) |  |  |  |  |
| At least 1 | 62.09 | 51.30 | 65.67 | 66.67 |
| Nicotine dependence† (%) |  |  |  |  |
| Low | 37.67 | 28.70 | 35.29 | 51.35 |
| Moderate | 41.86 | 39.13 | 46.08 | 36.93 |
| High | 20.47 | 32.17 | 18.63 | 11.71 |
| *Note:* Values represent mean (standard deviation) unless otherwise indicated. †Nicotine dependence was determined using the Heaviness of Smoking Index (HSI). Scores on the HSI range between 0 and 6; categories were formed based on previous research.^1^ | | | | |

**Table S3:** Baseline mixed logit model results

| Predictors | Estimate  (SE) | SD  (SE) |
| --- | --- | --- |
| Amount | 0.048 (0.002)*** | - |
| Cash | 0.331 (0.031)*** | 0.415 (0.052)*** |
| Vouchers | -0.331 |  |
| Healthcare centre | 0.127 (0.031)*** | 0.553 (0.046)*** |
| Workplace | -0.127 |  |
| Consistent | 0.067  (0.031)* | 0.127  (0.108) |
| Escalating | -0.067 |  |
| 1 every fortnight | 0.220  (0.056)*** | 0.392 (0.099)*** |
| 1 every week | 0.272  (0.059)*** | 0.294  (0.154)* |
| 2 every week | 0.114  (0.063) | 0.022  (0.364) |
| 3 every week | -0.606 |  |
| No programme^†^ | 1.227  (0.084)*** |  |
| *Notes:* 1. SE = standard error; SD = standard deviation; CI = confidence intervals.  2. **p* < .05; ***p* < .01; ****p* < .001.  3. ^†^a constant = 1 if “would not enrol in a programme” was selected or = 0 if the participant would enrol in their preferred programme.  4. Observations (N*tasks) = 4,300. Log Likelihood = -3,138.7 | | |

**References**

1. Borland R, Yong HH, O’Connor RJ, Hyland A, Thompson ME. The reliability and predictive validity of the heaviness of smoking index and its two components: Findings from the International Tobacco Control Four Country Study. *Nicotine Tob Res*. 2010;12(SUPPL. 1):45-50. doi:10.1093/ntr/ntq038
